# Restarting elective endoscopy safely amidst an evolving pandemic and the impact of patient perception

**DOI:** 10.1186/s12876-021-01917-z

**Published:** 2021-09-06

**Authors:** Christopher Nguyen, Kevin T. Kline, Shehzad Merwat, Sheharyar Merwat, Gurinder Luthra, Sreeram Parupudi, Steven Cohn

**Affiliations:** 1 Department of Internal Medicine, University of Texas Medical Branch, John Sealy Annex, 301 University BoulevardRoom 4.108, Galveston, TX 77555 USA; 2 Division of Gastroenterology and Hepatology, University of Texas Medical Branch, Galveston, TX USA

**Keywords:** COVID-19, Pandemic, Endoscopy, Re-opening, Patient perception

## Abstract

**Background:**

The COVID-19 pandemic has led to disruptions in elective and outpatient procedures. Guidance from the Centers for Medicare and Medicaid Services provided a framework for gradual reopening of outpatient clinical operations. As the infrastructure to restart endoscopy has been more clearly described, patient concerns regarding viral transmission during the procedure have been identified. Moreover, the efficacy of the measures in preventing transmission have not been clearly delineated.

**Methods:**

We identified patients with pandemic-related procedure cancellations from 3/16/2020 to 4/20/2020. Patients were stratified into tier groups (1–4) by urgency. Procedures were performed using our hospital risk mitigation strategies to minimize transmission risk. Patients who subsequently developed symptoms or tested for COVID-19 were recorded.

**Results:**

Among patients requiring emergent procedures, 57.14% could be scheduled at their originally intended interval. COVID-19 concerns represented the most common rescheduling barrier. No patients who underwent post-procedure testing were positive for COVID-19. No cases of endoscopy staff transmission were identified.

**Conclusions:**

Non-COVID-19 related patient care during the pandemic is a challenging process that evolved with the spread of infection, requiring dynamic monitoring and protocol optimization. We describe our successful model for reopening endoscopy suites using a tier-based system for safe reintroduction of elective procedures while minimizing transmission to patients and staff. Important barriers included financial and transmission concerns that need to be addressed to enable the return to pre-pandemic utilization of elective endoscopic procedures.

## Background

Since the first documented case of COVID-19 in the United States (US) on January 21, 2020, many hospitals have had to enact strict measures to prevent further spread of the virus among the population. The pandemic has led to widespread disruptions in the performance of elective and outpatient procedures. Endoscopic procedures were especially affected by these disruptions due to high rates of aerosolization of the virus by upper endoscopy, viral shedding in the stool, and potential contamination of endoscopy suite surfaces with viable virus up to 72 h after initial contact [1]. As endoscopic procedures also carry a high risk of viral spread to healthcare providers, it became necessary for hospitals to set up an organized model for re-opening endoscopy suites to elective procedures through a combination of testing, triage, and provision of appropriate personal protective equipment to patients and staff. Little is currently known regarding the efficacy of these measures in preventing transmission of the novel virus to staff and patients in this context. Since patient perception of procedure associated infection has been identified as a significant concern when restarting outpatient elective procedures, it is crucial to assess the safety of our procedures to ensure ongoing appropriate utilization of outpatient endoscopy [2].

As of the date of this manuscript, there have been over 33 million confirmed cases of COVID-19 in the United States out of 173 million cases worldwide with over 3.5 million COVID-19 related deaths worldwide [3]. The infectious transmission of the virus is thought to be via respiratory droplets with potential for airborne spread during aerosol-producing procedures, such as endoscopy [4]. The insidious nature of the epidemic stems from its high rate of asymptomatic spread, with 80% of cases being mild or asymptomatic [4]. Considering this, the US Surgeon General recommended the cancellation of all elective procedures on March 14, 2020 [5]. Soon after on March 17, 2020, the American College of Surgeons, American Gastroenterological Association (AGA), and the other national gastroenterology societies recommended rescheduling elective procedures to focus only urgent and emergent endoscopic procedures [6–8].

The first inpatient case of COVID-19 at our institution was confirmed on March 3, 2020. As the number of inpatient cases increased, the capacity to safely perform invasive procedures decreased with limited guidance regarding appropriate re-opening strategies and stratification of endoscopic cases from the joint GI societies [9, 10]. As the prevalence of disease became more clearly established by widespread testing within our institution and community, the Division of Gastroenterology and Hepatology developed a tiered strategy in an effort to resume timely scheduling of patients awaiting diagnostic procedures. The Centers for Medicare and Medicaid Services (CMS), in concordance with multi-society guidelines, helped provide a framework for gradual reopening of outpatient clinical procedures. Here we describe the process and outcomes of a tier-based system for scheduling outpatient endoscopy, in the context of an ongoing, and evolving pandemic, to provide diagnostic and screening endoscopy services within a reasonable period applying appropriate risk mitigation precautions for patients and staff members. In addition, within the context of those needing urgent procedures we identified barriers to proceeding with endoscopy. Despite the absence of procedural COVID-19 transmission at our institution, concerns voiced by patients provided important insight into challenges the gastroenterology community will have to address in the immediate future and beyond.

## Methods

Using the EPIC electronic medical record (EMR), patients with cancelled procedures due to the COVID-19 pandemic from 3/16/2020 to 4/20/2020 were identified from two separate practice locations within our medical system, the University of Texas Medical Branch (UTMB) endoscopy unit in Galveston and the UTMB League City campus endoscopy unit. Patients were stratified into tier groups (1–4) by gastroenterology faculty in order of urgency (Table 1). During phase 1 of Texas’s reopening, tier 1 (urgent/emergent) and tier 2 (semi-urgent) cases were identified and reviewed by the newly formed Endoscopy Committee and, if approved, were contacted by the nursing staff and scheduled within 1 week and 4 weeks respectively. During phase 2, tier 3 and 4 cases were to be rescheduled within 3 months. After the initial scheduling, the barriers to successful rescheduling were assessed and organized by the nursing staff (Table 2). Within a short period after the initiation of the tier system, once the urgent and semi-urgent cases were scheduled, due to increase surge capacity all tiers were eventually accommodated at the patient’s earliest convenience.

**Table 1:** Tier system

Tier	Criteria	Timing
1	Abnormal imaging, rapid weight loss, dehydration due to GI symptoms, ongoing GI bleeding, acute dysphagia	Within 1 week
2	Chronic diarrhea, chronic dysphagia, chronic anemia, Post-polypectomy/resection follow up, variceal screening with history of bleed	Within 4 weeks
3	Barrett’s follow up/surveillance, variceal screening without bleed, IBD surveillance	Within 3 months
4	Screening or surveillance procedures for polyps without any alarm signs	After 3 months

Tier system to prioritize rescheduling outpatient endoscopy procedures consisting of the criteria for each tier and their respective rescheduling time frame goals

**Table 2:** Endoscopy rescheduling due to COVID-19 pandemic

Tier	1 (n = 16)	2 (n = 170)	3 (n = 140)	4 (n = 214)
**Total reached by phone**	14 (87.50%)	147 (86.47%)	105 (75%)	120 (56.07%)
**Total rescheduled**	8	72	62	85
**Rescheduling timing**				
Within 1 week	2	51	17	5
Within 4 weeks	5	19	42	63
After 4 weeks	1	2	3	17
**Reasons for not rescheduling**				
COVID-19 concerns	3	30	30	25
Financial	0	6	4	0
Transport	0	9	3	0
Other	3	30	15	7
COVID-19 positive	0	1	0	0

Number of patients successfully rescheduled within their respective time frame goals in addition to patients who deferred rescheduling and the reasons for deferment

Our hospital risk mitigation strategies included a strict no visitor policy, temperature and symptom screening of all hospital employees at the entrance to the hospital, mandating the use of masks in all patient care areas of the hospital, maintaining 6 feet of social distancing, and mandatory testing of all inpatients for COVID-19 as well as COVID-19 testing for all health care providers involved in endoscopy patient care. In the endoscopy unit, comprehensive personal protective equipment (PPE) protocols were developed based on national and international guidance and experience including: head coverings, gowning, N95 masks for all staff during endoscopy (with mandate for prior fit testing performed) in addition to an overlying surgical mask, eye protection, and double gloving. Specific doffing and donning techniques with hand hygiene were implemented and quality checks by staff were enforced [11, 12]. Enhanced cleaning techniques were implemented for each active endoscopy suite between cases, with increased time between scheduled cases to account for these measures. All patients underwent pre-procedure COVID-19 testing within 72 h of their scheduled procedure with polymerase chain reaction (PCR) test, or rapid testing on day of procedure, if unable to be screened prior. Both were administered and processed at our institution. In addition, all patients were screened for COVID-19 symptoms on the day of procedure at admission with a plan to reschedule anyone with a positive COVID-19 test or suggestive symptoms.

Patients who had completed their respective endoscopic procedures were contacted by telephone to inquire if they underwent COVID-19 testing within 14 days post-procedure (Table 3). The symptoms experienced at the time, reason for testing, and results of the testing were assessed to evaluate the efficacy of peri-procedural transmission prevention measures.

**Table 3:** Pre and post-procedure COVID-19 testing

Procedure	Rapid ID	PCR	Days before procedure	Fever	Cough	SOB	Anosmia/ Ageusia	GI symptoms	Responded to survey	Postop testing	n
EGD	92.4% (61)	7.6% (5)	2.0 days	3.0% (2)	13.6% (9)	7.6% (5)	1.5% (1)	7.6% (5)	90.9% (60)	25.8% (17)	66
ERCP	100% (11)	0	1.2 days	0	0	9.1% (1)	0	27.3% (3)	72.7% (8)	27.3% (3)	11
EUS	100% (2)	0	2.0 days	0	50% (1)	50% (1)	50% (1)	0	100% (2)	0	2
EUS/ERCP	83.3% (5)	16.7 (1)	1.7 days	0	33.3% (2)	16.7 (1)	0	0	50% (3)	16.7 (1)	6
Enteroscopy	100% (1)	0	1	0	100% (1)	100% (1)	0	0	100% (1)	0	1
PEG	(1)	0	1						0	0	1
Total Upper Endoscopies	93% (81)	7% (6)	1.8	2.3% (2)	14.9% (13)	10.3% (9)	2.6% (2)	9.2% (8)	85.1% (74)	24.1% (21)	87
Colonoscopy	94.7% (124)	5.3% (7)	2.1 days	0.8% (1)	1.6% (2)	0.8% (1)	0	3.1% (4)	88.5% (116)	3.8% (5)	131
Ileoscopy	100% (1)	0	2	0	0	0	0	0	100%	0	1
Flex sig	100% (2)	0	2	0	0	0	0	0	50% (1)	0	2
Total Lower Endoscopies	96.3% (129)	4.0% (7)	2.1	0.7% (1)	1.5% (2)	0.7% (1)	0	3.0% (4)	87.3% (117)	3.7% (5)	134
EGD/Colon	96.7% (58)	3.3% (2)	2.1 days	1.67% (1)	10% (6)	6.7% (4)	0	1.67% (1)	73.3% (44)	18.3% (11)	60
All	94.7% (266)	5.3% (15)	2.0	1.4% (4)	7.5% (21)	5% (14)	0.7% (2)	4.6% (13)	87.5% (246)	13.2% (37)	281

Total procedures performed stratified by type of procedure. Pre-operative and post-operative COVID-19 test results were recorded

## Results

Of the 540 patients awaiting delayed endoscopic procedures between 3/16/2020 to 4/20/2020 due to the pandemic, 227 patients across all tiers were rescheduled. Of these, only 57.14% of tier 1 and 48.97% of tier 2 could be scheduled at their originally intended procedure interval, respectively. Even with the capability to accommodate for all tier 1 and tier 2 patients, the average delay of endoscopy from the initial scheduled procedure date to index endoscopy was 32 days, with the longest delay being 86 days. Despite counseling on extensive safety measures instituted as well as the indication for the procedure, the most identified barrier for patients to return to undergo urgent or semi-urgent endoscopy was COVID-19 related concerns in 40% of tier 1 and tier 2 patients. 7.2% of patients in the same cohort identified new financial limitations as reasons for not scheduling. Patient refusals while scheduling Tiers 1 & 2 allowed prompt scheduling of majority of the other tiers early. Our pre-procedure COVID-19 testing of the patients willing to return for their procedures revealed a seroprevalence of 1.3%, which is lower than the test positivity of 4.4% in the general population of Galveston county at the same time and continued to rise even after the conclusion of our study.

Of the 281 patients who underwent their re-scheduled endoscopic procedures (Table 3), 94.7% of patients received the rapid molecular diagnostic test (Rapid ID Now) and 5.3% of patients received the PCR test. The average number of days patients were tested prior to their procedures was 2 days. 24% of patients who underwent upper endoscopies and 3.7% of those who underwent lower endoscopies were tested again during post-procedure follow up either due to new-onset symptoms suspicious for COVID-19 infection, or requiring hospitalization for unrelated reasons, or needing medical clearance prior to follow-up procedure. Of the patients who were symptomatic, fever, cough and gastrointestinal symptoms were the most reported. None of the patients who underwent post-procedure testing were positive for COVID-19. Of the 4 patients that reported fever after endoscopy, 3 of the patients tested negative for COVID-19 by rapid assay. One patient declined testing because fever resolved within 24 h. During the study period, no cases of endoscopy unit transmission to staff were identified.

Of the patients in tier 1 and tier 2 who successfully underwent rescheduled endoscopy, 4 patients were diagnosed with a gastrointestinal malignancy or inflammatory bowel disease. These cases consisted of gastric adenocarcinoma, esophageal adenocarcinoma, ulcerative colitis, and Crohn’s disease. The average delay of endoscopy in these 4 patients was 34 days from the initial scheduled procedure date to the index endoscopy. The patient with gastric adenocarcinoma passed away within 1 month of diagnosis. The patient with Crohn’s disease was listed as deceased within 3 months of diagnosis for unclear reasons and occurred outside the confines of our electronic medical record. No significant interim upper or lower gastrointestinal bleeds were identified.

## Discussion

In an attempt to preserve hospital beds, resources, PPE and to protect staff and patients during the COVID-19 pandemic, non-emergent endoscopies were delayed or canceled leading to a substantial reduction in procedures performed in endoscopy suites. Use of our model for re-opening endoscopy suites by a tier-based system allowed recognition of barriers for scheduling and facilitated appropriate utilization of the excess capacity to accommodate most patients across all tiers within 4 weeks. Thus, minimizing delays in patient care.

By practicing our strict risk mitigation strategies, which were in line with experiences and practice in Italy implicating low risk of transmission during endoscopy if the proper precautions are taken, we were able to limit the peri-procedure transmission to nil as shown by the data from our post-procedure surveys (Table 3) [13, 14]. Despite these successes, the barriers to restarting endoscopy in our study remain the public perception of safety in addition to the financial cost and loss of insurance coverage during the pandemic. This narrowed the utility of our tier system, as our highest priority patients identified COVID-19 related unemployment and financial limitations besides peri-procedure infection concerns as reasons for postponing their procedures. While COVID-19 related concerns accounted for the largest proportion of patients in all groups, a financial disparity was noted in comparison to our Tier 4 patients (most often average risk colon cancer screening colonoscopies), none of whom reported insurance or cost concerns for reasons for postponement. Interestingly, our reported rates of COVID-19 concern related postponement in procedures are much higher than the rates reported by Rex et al., where only 4.3% were unwilling to undergo elective endoscopy in May 2020 versus 16.3% across all tiers in our cohort [2]. Partly this difference is likely related to relative lower socioeconomic status of our main hospital’s catchment area, Galveston County, including a 12.9% poverty rate prior to the pandemic. Individuals with lower incomes and education perceive higher personal health and financial threats from COVID-19 compared to other groups [15]. This may explain this disparity, particularly at the beginning of the pandemic when infection burden in our area was relatively low compared to other parts of the country. Relatively slower peaking of the infection rates in the region led to a situation where public perception and fear played a role in some delay in resuming the procedural services. Implementation of safety measures with a robust testing capacity and easy access for patients and endoscopy staff as well as appropriate planning for adequate supplies of PPE from the onset and throughout allowed relatively high overall number of patients to eventually undergoing endoscopic procedures during this period. While the tiered approach for rescheduling and higher patient response rate for survey after the procedures are the strengths of the study, the limitations include relatively lower number of Tier 1 and Tier 4 patients who could be rescheduled. After initial testing, lack of serial formal COVID-19 testing for endoscopy staff during the study period is another potential limitation to detect asymptomatic transmission.

Suspending non-urgent endoscopic procedures has serious clinical and financial implications. Approximately 18 million endoscopic procedures are performed annually in the US with an estimated annual healthcare cost of $32.4 billion [16, 17]. The consequences of suspending elective endoscopy for even 6 months has grim medical implications including the impediment of surveilling nearly 1,500 fatal but preventable variceal bleeds in cirrhotic patients [18–20]. Additionally, there could be a delay in the diagnosis of nearly 22,000 high grade adenomatous polyps with malignant potential and 3000 colorectal cancers, resulting in a 6.5% increase in the 6-month mortality rate for patients who would eventually be diagnosed with colorectal cancer [17, 21, 22]. Our study identified delays in the diagnosis of 2 gastrointestinal malignancies and 2 cases of inflammatory bowel disease in patients with the highest priority in endoscopy. A recent study estimated a 58% decrease in weekly cancer detections and up to a 95% decrease in endoscopic activity during the height of the COVID-19 pandemic when compared to pre-COVID-19 activity [23]. It is therefore important to methodically recommence non-emergent endoscopy to minimize the economic and health impacts of its suspension.

With the progression of the COVID-19 pandemic, multiple best practice guidelines have emerged regarding pre-procedure, peri-procedure, and post-procedure risk mitigation strategies. One particular study analyzed the consensus of effectiveness of these strategies among multi-national large endoscopy departments using a modified Delphi method [24]. Many of the high consensus recommendations were in-line with the mitigation strategies performed at our institution. Other recent studies have detailed the most cost-effective strategies of pre-procedure testing and risk stratification including similar use of telephone screening methods as in our study [24, 25].

To conclude, our tiered approach for rescheduling while following strict risk mitigation strategies allowed for successfully resuming outpatient endoscopic procedures keeping patients as well as endoscopy staff safe and protected from COVID-19 transmission. Patients who opted to proceed with their procedures were successfully accommodated with 15 min additional time allotted to room cleaning and turnover per case. Additionally, our mitigation measures resulted in no transmission of COVID-19 cases that could be traced back to exposure during endoscopy. Future studies regarding procedural transmission of COVID-19 in the asymptomatic population may further allay patient fears. However, the financial impact of this pandemic will continue to be a stubborn remnant from this novel virus, delaying patient care and impacting utilization of endoscopy in the future.

## Conclusion

By implementing a tiered approach to reschedule patients undergoing elective endoscopy during the height of the COVID-19 pandemic with strict risk mitigation strategies, our institution was able to safely restart outpatient endoscopy to minimize delays in endoscopic diagnosis and patient care. All patients deemed higher risk that were willing to undergo endoscopy were accommodated for. The main barrier to rescheduling was public perception of safety. The risk mitigation measures taken allowed for zero transmission of COVID-19 among patients and staff members.

## Data Availability

The patients in our study have not been reported in any other submission. The data can be accessed via the links to a repository provided below: https://figshare.com/s/e63ad2152c81c9f8abe4, https://figshare.com/s/68fef52c0c03804f05f3, https://figshare.com/s/770d244fa95c70332737.

## Abbreviations

AGA: American Gastroenterological Association
CMS: Centers for Medicaid and Medicare Services
EGD: Esophagogastroduodenoscopy
EMR: Electronic Medical Record
GI: Gastroenterology
PCR: Polymerase Chain Reaction
PPE: Personal Protective Equipment
US: United States
UTMB: University of Texas Medical Branch
